# The Application of GSCM in Eliminating Healthcare Waste: Hospital EDC as an Example

**DOI:** 10.3390/ijerph16214087

**Published:** 2019-10-24

**Authors:** Huan-Cheng Chang, Mei-Chin Wang, Hung-Chang Liao, Ya-huei Wang

**Affiliations:** 1 Division of Nephrology, Department of Medicine, Landseed International Hospital, No. 77, Guangtai Road, Pingzhen Dist., Taoyuan 324, Taiwan; changhc@landseed.com.tw; 2 Department of Health Care Management, Chang Gung University, No. 259, Wenhua 1st Road, Guishan Dist., Taoyuan 33302, Taiwan; 3 Noble Health Management Center, Landseed International Hospital, No. 77, Guangtai Road, Pingzhen Dist., Taoyuan 324, Taiwan; wangmeria@landseed.com.tw; 4 Department of Medical Management, Chung Shan Medical University Hospital, No. 110, Section 1, Jian-Koa N. Road, Taichung 402, Taiwan; 5 Department of Health Services Administration, Chung Shan Medical University, No. 110, Section 1, Jian-Koa N. Road, Taichung 402, Taiwan; 6 Department of Applied Foreign Languages, Chung Shan Medical University, No. 110, Section 1, Jian-Koa N. Road, Taichung 402, Taiwan

**Keywords:** green supply chain management (GSCM), emergency medicine service (EMS), emergency department crowding (EDC), dynamic Taguchi method, neural network (NN), genetic algorithm (GA)

## Abstract

Eliminating unnecessary healthcare waste in hospitals and providing better healthcare quality are the core issues of green supply chain management (GSCM). Hence, this study used a hospital’s emergency department crowding (EDC) problem to illustrate how to establish an emergency medicine service (EMS) simulation system to obtain a robust parameters setting for solving hospitals’ EDC and waste problems, thereby increasing healthcare quality. Inappropriate resource allocation results in more serious EDC; more serious EDC results in increasing operating costs. Therefore, in the healthcare system, waste includes inappropriate costs and inappropriate resource allocation. The EMS of a medical center in central Taiwan was the object of the study. In this study, the dynamic Taguchi method was used to set the signal factor, noise factor, and control factors to simulate the EMS system to obtain the optimal parameters setting. The performance was set to Emergency Department Work Index (*EDWIN^C^*) and system time (waiting time and service time) per patient. The signal factor was set to the number of physicians; the noise factor was set to patient arrival rate; the control factors included persuading Triage 4 and Triage 5 outpatients, checkup process, bed occupation rate in the emergency department (ED), and medical checkup sequence for Triage 4 and Triage 5 patients. This study makes two significant contributions. First, the study introduces the GSCM concept to the healthcare setting to bring green innovation to hospitals. Hospital administrators may hence design better GSCM activities to facilitate healthcare processes to provide better healthcare outcomes. Second, the study applied the dynamic Taguchi method to the EMS and neural network (NN) to construct a computational model revealing the cause (factors) and effect (performances) relationship. In addition, the genetic algorithm (GA), a solution method, was used to obtain the optimal parameters setting of the EDC in Taiwan. Hence, after obtaining the solutions, the unnecessary waste in EDC—inappropriate costs and inappropriate resource allocation—is reduced.

## 1. Introduction

In the environmental and public health field, many scholars in recent years have studied green supply chain management (GSCM). GSCM emphasizes the reduction and control of environmental impacts, such as pollution from production processes or products, waste of resources, and overproduction [1]. Hence, decision-makers take this important problem into consideration when designing and manufacturing products or providing services.

The healthcare systems’ GSCM can facilitate hospitals’ environmental management practices. Thus, GSCM of a healthcare system will not increase process costs, disrupt the flow of existing processes, or cause environmental problems [2,3]. Considering GSCM in the healthcare system, the protection of patients’ health is the most important factor [4] because patient health is a hospital’s core value. However, little research has been done on healthcare GSCM. de Oliveira et al. [5] reviewed 194 academic journal articles from the last ten years on the implications and future directions of GSCM. Most papers covered the industrial sectors of textiles/manufacturing, automotive, multiple industrial sectors, and electronics. Only three papers [6,7,8] address the health (beauty and hygiene) and medical sectors. Even after 2016, little research had been undertaken regarding healthcare GSCM. Sohjaie et al. [9] used the fuzzy ELECTRE method to analyze and sort the green suppliers for a green health supply chain. An Iranian pharmaceutical company was the research case. Given this dearth of research, there is a great opportunity for future research in healthcare systems’ GSCM [5]. Hence, the researchers felt the necessity to use GSCM to eliminate healthcare waste. Furthermore, to solve a hospital’s emergency department crowding (EDC) problem, the researchers wonder about the possibility of using the concept of GSCM to set up an emergency medicine service (EMS) system to optimize resource allocation to solve hospitals’ EDC and waste problems.

## 2. Literature Review

Hospitals generally play a key role in hospital supply chain management (HSCM) because hospitals directly face the customers (patients), as their main role is to provide healthcare services to patients. However, unnecessary healthcare waste, including inappropriate costs and resource allocation, may decrease healthcare quality. Therefore, in the study, the “green” in GSCM focuses on resource utilization to reduce hospitals’ operation costs. Muduli et al. [10] think that capacity constraints and resource utilization are the major barriers to the implementation of GSCM. To research “improving capacity utilization” in GSCM and evaluate the best-performing organizations, Sari [11] explored a novel multi-criteria decision framework, which includes GSCM’s performance and capacity utilization. Wu et al. [12] considered how to manage financing risks when the capacity is restricted for increasing the competitive strength of GSCM. Wang et al. [13] analyzed different price models for the effect on GSCM when considering capacity constraint.

In the development of GSCM in service industries, Liu and Chen [14] created a dyadic model of GSCM that considers the profit of a news vendor retailer. The retailer’s behavior is based on Kirshner and Shao’s [15] GSCM mathematical model, which is used to solve problems in service industries. Coskun et al. [16] considered the gap between service life cycles and consumers’ expectations to establish mathematical models that measure the relationship between GSCM and consumer behaviors. Based on the development of GSCM in service industries, this paper extends the concept of GSCM to hospital departments and focuses on the utilization of healthcare resources to avoid unnecessary healthcare waste and increase healthcare quality. When hospital administrators consider GSCM, they may design better GSCM activities between the input and output healthcare services that facilitate smoother healthcare processes and better healthcare quality.

In considering facilitating smoother healthcare processes and better healthcare quality, scholars over the last two decades have regarded overcrowding and inadequate capacity of Emergency Departments (EDs) as an urgent public health problem [17]. An Emergency Room (ER) provides a specialized medical service that is open year-round; it provides critical medical care for patients urgently in need. Therefore, ERs should always be ready to handle emergency and non-emergency situations to provide comprehensive critical care. However, ER medical professionals must handle high volume and unpredictable patient flow and workloads, which leads to an excessive need in the internal control of an ER [18]. Studies have shown that most patients are dissatisfied with their ER’s care [19,20]. Overcrowding can result in an extended stay for a patient. The risk of mortality—for example, increased complications for myocardial infarction—is increased by an extended length of stay (LOS) [21,22]. Therefore, shortening a patient’s stay in an ER will improve the quality of ER care [23]. One of the problems with ER congestion is that it may endanger a patient’s safety if the patient leaves the hospital without obtaining a physician’s diagnosis and treatment. When congestion occurs, patients’ lengths of stay become longer. When LOS becomes longer, the probability of a patient leaving without a diagnosis or treatment is higher [24].

In an ED, the length of hospital stay is an important criterion for measuring the quality of care [25]. Asaro et al. [26] discovered that the congestion factor in the ED elongates waiting time and hospitalization time. Rathlev et al. [27] found that the time spent in the ER is positively correlated with the hospitalization admission rate. A major limitation in the studies conducted thus far is the use of static methods to measure ER congestion, either discussing the ER congestion at a point in time (e.g., a patient’s arrival time) or during a specific time interval (e.g., transferring). However, many studies have indicated that ER congestion belongs to dynamic congestion. There is substantial fluctuation in the period (LOS) a patient remains in the ED [28,29,30].

Many strategies have been proposed to solve the ER congestion problem [31]. Providing timely patient care to reduce LOS may ease the overcrowding in the ER and hence, increase patient safety and improve healthcare quality [32]. Scholars have also considered ambulance diversion to reduce ER congestion. Ambulances are usually called to divert patients to nearby hospitals to avoid congestion. When an ED expects that it has insufficient capacity to accept new patients, ambulance diversion becomes a common phenomenon to avoid delays in patient transfer and the loss of critical time for treatment [33]. Many institutions, such as the Emergency Nurses Association and the American College of Emergency Physicians, have suggested how to improve patient flow control to reduce ER congestion [34]. The operational efficiencies in the management of ED patient flow can be used to alleviate ED congestion. However, some studies have revealed that hospital mangers may resort to the control of patient flow to reduce ED congestion, not intending to invest in the relatively higher cost in ED facilities, equipment, and professionals to improve healthcare quality [35,36]. To eliminate EDC, hospital administrators should consider wait times, layouts of facilities, lengths of hospitalization, etc., to increase the quality of patient care.

Many studies have used different mathematical methods to solve the problem of EDC. Grekousis and Liu [37] modeled emergency events and used a neural network (NN) to forecast the demand on EDs. They also applied a new evolutionary algorithm to improve location plan and decision making. Goto et al. [38] created an artificial intelligence model to select patients who require urgent revascularization within forty-eight hours of medical treatment. Xia et al. [39] used big data from global position systems to evaluate medical accessibility in EMS to evaluate the effectiveness of public health services. de Oliveira et al. [40] developed emergency care delivery models that may apply a routing algorithm to select the optimal EMS for highly complex patients.

Based on the above-mentioned literature review, this study aimed to attempt the following: (1) to construct a system for the EMS, (2) to reduce EDC based on the constructed EMS system, (3) to obtain better service quality. The attempts were all involved in the research issue of GSCM in the healthcare system.

## 3. Research Objectives

Eliminating unnecessary healthcare waste in hospitals and providing better quality healthcare are the core issues of green supply chain management (GSCM). To eliminate unnecessary healthcare waste and solve the EDC problem to provide better quality healthcare, this study set up an EMS simulation system to obtain a robust parameters setting; internal medical patients at the ED of a medical center in central Taiwan were the objects of the study. A statistical calculation of the healthcare resource allocation of each procedure using each patient’s actual time spent during each medical procedure was performed. A simulation system of the emergency procedures for the ED patients was also created, including the number of people at each triage, the ED procedures, and the time spent for each procedure. Different improvement strategies were introduced and applied to the simulation system to derive an appropriate allocation of the healthcare resources, to find the optimal combination of the improvement strategies, to shorten the time spent by patients in the ED, to alleviate the level of EDC, and to enhance the operational efficiency of the emergency department. 

The study used the dynamic Taguchi method to build a simulation system for the EMS, in which NN was used to construct a computational model revealing the cause (factors) and effect (performances) relationship. Finally, a solution method, the genetic algorithm (GA), sets the optimal parameters (the optimal combination of improvement strategies) for the optimal performances in the EMS system. The purpose of this study was to help hospital administrators apply the concept of GSCM to eliminate unnecessary healthcare waste using EDC as an example and hence, provide better quality healthcare.

## 4. Setting Factors Levels in the EDC

To use GSCM to eliminate unnecessary healthcare waste, the author used the dynamic Taguchi method to set the signal factor, noise factor, and control factors, to simulate the EMS system to obtain the optimal parameters setting. The signal factor was set to the number of physicians, the noise factor was set to patient arrival rate, and the control factors included persuading Triage 4 and Triage 5 outpatients, checkup process, ED bed occupation rate, and medical checkup sequence for Triage 4 and Triage 5 outpatients. The research scope was the internal medicine patients of the EMS system.

The signal factor was the number of physicians. Here, the number of physicians could be set to 1 or 2 persons, depending on the EDC situation. The patient arrival rate was divided into two situations: one was the regular day situation, and the other was the holiday situation. The Poisson distribution was $P(x)=\frac{\lambda^{x}}{x!}e^{-\lambda }$, in which λ was the patient arrival rate $\frac{\mathrm{pateints}}{\mathrm{day}}$, and x was the number of patients each day. Exponential distribution was applied here for transfer Poisson distribution to obtain x. Exponential distribution was defined as $f(x)=\lambda e^{-\lambda x}$. F(x) was the cumulative distribution function of f(x). This study used F(x)=θ (θ was simulated using the Monte Carlo method; the value was between 0 to 1 to obtain the $x_{t}$, in which $x_{t}$ was the number of patients in period t). Because outpatient services are closed on holidays, a patient must go to the ED. So the researchers set different patient arrival rates for regular days and holidays. Hence, the noise factor was the patient arrival rate. Here, Level 1 was set to 350, and Level 2 was set to 425.

There were three levels set for control factor A, persuading Triage 4 and Triage 5 outpatients. Level 1 was in the situation when $1.5\leq EDWIN^{C}$ of the previous day <2 (EDWIN: Emergency Department Work Index). Level 2 was the situation when $2\leq EDWIN^{C}$ of the previous day <2.5. Level 3 was the situation when $2.5\leq EDWIN^{C}$ of the previous day. $EDWIN^{C}$ is described as follows: $EDWIN^{C}$ was from EDWIN. An EDWIN was created to represent patient triage units per available bed for each physician on duty. Research [28,29,41,42] has shown that a medical staff’s perception of busyness is significantly related to the EDWIN score, and that EDWIN demonstrates a good validity on the prediction of EDC.

In this study, surveys were distributed to the ER medical staff to obtain the busy hours and periods in the ER. The results derived from the surveys were combined with EDWIN for statistical analysis. Equation (1) shows EDWIN.

(1) $$EDWIN=\frac{\sum n_{i}t_{i}}{N_{a}(B_{t}-B_{A})}$$

$n_{i}$: number of patients at each triage;

$t_{i}$: level of triage (i=5 refers to the most urgent patient);

$N_{a}$: number of physicians on duty at the ED;

$B_{t}$: treatment beds that are registered at ED (excluding all kinds of beds in the lobby and in the corridors);

$B_{A}$: number of patients admitted.

An EDWIN value lower than 1.5 indicates that the ER is in good condition; a value between 1.5 and 2 indicates that it is in a busy condition; a value over 2 indicates EDC.

However, according to Equation (1), i=5 shows the highest emergency situation, while in Taiwan, according to Taiwan Triage and Acuity Scale (TTAS), i=1 shows the highest emergency situation. Hence, in this study, the EDWIN should be transformed into $EDWIN^{C}$, as in Equation (2).

(2) $$EDWIN^{C}=\frac{\sum n_{i}(6-i)}{N_{a}(B_{t}-B_{A})}$$

The control factor B was the checkup process. There were three levels. Level 1 was in the situation in which emergency patients had the priority to use the exam rooms; the enforced procedure was urine→ CT →X-ray for the exam. Level 2 was the situation in which emergency patients had the priority to use the exam rooms, but the above procedure was not enforced; if the exam room was occupied, the patient could move on to the next exam room for the exam. Level 3 was the situation in which the patient had the choice to randomly select the exam room. The control factor C was the ED bed occupation rate. There were three levels. Level 1 was the situation in which more than 15% (>15%) of ED patients were waiting for sickbeds, and a maximum quota of five empty beds in the internal medicine department could be made available for emergency patients. Level 2 was the situation in which more than 20% (>20%) of ED patients were waiting for sickbeds, and a maximum quota of ten empty beds in the internal medicine department could be made available for emergency patients. Level 3 was the situation in which more than 25% (>25%) of ED patients were waiting for sickbeds, and a maximum quota of fifteen empty beds in the internal medicine department could be made available for emergency patients.

The control factor D was the checkup sequence for Triage 4 and Triage 5 outpatients. There were 3 levels. Level 1 was the priority treatment given to Triage 4 and Triage 5 outpatients, who required shorter examinations. Level 2 was the routine checkup sequence for Triage 4 and Triage 5 outpatients; that is, the Triage 4 and Triage 5 outpatients were examined according to the sequence of registration. Level 3 was the checkup sequence, with Triage 4 outpatients diagnosed first and then Triage 5 outpatients.

The system performances were considered based on the concept of the Taguchi loss function. The Taguchi loss function is a landmark in improving service quality [43]. Inappropriate services will result in patients’ dissatisfaction. Thus, patients’ dissatisfaction will be spread and result in some loss to hospitals, patients, or the wider community. These losses are defined as social loss. To avoid the loss, hospitals seek to enhance their reputation. A way to eliminate such social costs is to improve service quality, as in the study, to improve the service index in the system. The system time for each patient, which included the waiting time and service time, and $EDWIN^{C}$ was considered because these two service indexes directly bring impacts on the service quality. In addition, many researchers have attempted to solve the EDC problem, emphasizing decreasing patients’ stay in the systems and $EDWIN^{C}$ [44,45,46]. Equation (3) shows the formulation of the system time (ST). Equation (2) shows the other performance, $EDWIN^{C}$.

(3) $$ST=\sum_{i=1}^{m}W_{i}+\sum_{j=1}^{n}S_{j}$$

$W_{i}$: the waiting time for the *i*th waiting room;

$S_{j}$: the service time for the *j*th service station.

## 5. The Dynamic Taguchi Method and Neural Network for the Optimal Factors Levels Setting

To obtain the optimal parameters setting, four steps, including dynamic method and neural network, were used to achieve the research objectives.

### 5.1. STEP 1. Use the Orthogonal Array of the Taguchi Method to Derive the Simulation Data

This study used the orthogonal array of the Taguchi method to obtain the simulation data. Ahalt et al. [44] used a discrete-event simulation approach to compare different EDC’s scores in a large academic hospital in North Carolina to provide some strategies to bring better patient care outcomes. Hurwitz et al. [47] created a flexible simulation platform to quantify the EDC’s information, which can be used in the management of EDC. Hoot et al. [48] developed a simulation approach for EDC to forecast the near future situation. The forecasting results showed that the actual outcomes are near to the forecasting results. Using simulation data to solve EDC problems has been used by an increasing number of researchers. However, these simulation scenarios are case by case. Hence, the selected parameters for constructing the simulation scenario must be considered based on the selected hospitals [49]. 

Furthermore, to evaluate robustness and variation in the EMS system, a signal-to-noise (SN) ratio was used in the system. A higher SN value corresponds to better performance and less response variation [43].

Using the dynamic Taguchi method to resolve the EMS problem, the EMS formed a dynamic multi-response system including two responses, two signal levels, four control factors, and two noise factor levels. Regarding the two responses, the response Y was determined by a set of signal settings $M=(M_{1},M_{2})$, a set of control factor vectors $X=(X_{1},\ldots ,X_{4})$, and a set of noise factor settings $Z=(Z_{1},Z_{2})$. The response model of the EMS system could be indicated as Y=f(M,X,Z)+ε, in which ε was the error term. Hence, in the formed dynamic EMS system,$y_{ij}$ denoted the *i*th response corresponding to the *j*th signal factors level. Assuming there was no intercept in the linear ideal function, there came to $y_{ij}=\beta M_{j}$, where $M_{j}$ denoted the *j*th signal factors level and β was the slope. To evaluate the performance in the dynamic EMS systems using the Taguchi method, the equation $SN=10\log_{10}(\beta /MSE)$ was applied, in which *MSE* was the mean square error of the distance from the measured response to the best-fitted line [50,51].

The orthogonal array, $L_{9}$, was simulated to obtain the performance data. Thirty-six, 2*2*9 (2 levels for noise factor, 2 levels for signal factors, and control factors for $L_{9}$), combinations of parameter levels were simulated. Each combination was simulated 1000 times. The results showed that based on the performance of $EDWIN^{C}$, the linear ideal function was Y=1.87−0.5M+error, the optimal parameters setting for factors (A, B, C, D) was (Level 1, Level 1, Level 1, Level 1) and the expected result at optimum condition was 11.04 dB (dB is a unit of measurement for SN). In addition, the result showed that based on the performance of ST, the optimal parameters setting for factors (A, B, C, D) was (Level 3, Level 3, Level 2, Level 2) and the expected result at optimum condition was 1.01 dB.

### 5.2. STEP 2. Build the Relationship between Parameters and Performances

Because the time variables were a probability distribution, this study used the NN to construct a mathematical model to obtain the optimal parameters setting for the EMS. The input nodes for the NN were the signal factor levels, noise factor levels, and control factors levels, and the output nodes for the NN were desirability values $d_{1}$ from normalized ST and $d_{2}$ from normalized $EDWIN^{C}$. The NN’s procedure is as follows: 

A soft computing algorithm, NN, has been used as a computational model in different research fields, such as medical diagnoses, financial analysis, signal processing, and pattern recognition. Inspired by animals’ central nervous systems, an NN was created to mimic a human biological, neurological network. The network is formed by parallel processing units, that is, neurons or nodes. The nodes are linked together to form a network, in which knowledge is derived through the interconnection or relationship between input and output nodes. The weighted and transformed function is used for the weighted sum of the previous input neuronal layers, except the first layer. Different weighted and transformed functions used in the NN meant that NNs could be used in wide-ranging applications. Therefore, NNs create a black-box mathematical model, with a form of nonlinear mathematical network structure. That is, if an NN’s architecture and parameters can be adequately selected, an NN can effectively address complex nonlinear problems [52,53]. 

The normalized ST and $EDWIN^{C}$ were used as desirability functions. This study initially evaluated the performance of each dynamic response by using the modified desirability functions [54]. The desirability functions were normalized by an estimated response ${\hat{y}}_{i}$ according to ST and $EDWIN^{C}$. The exponential functions were then used to transform the normalized value to a scale-free value *d_i_*, called desirability. This normalized value was between 0 and 1, which increased as the desirability of the corresponding response increased. In the responses of the EMS system, ST was STB (smaller-the-better), while $y_{2}$($EDWIN^{C}$) was LTB (larger-the-better). Equations (4) and (5) define the desirability values $d_{1}$ and $d_{2}$.

(4) $$d_{1}=\{\begin{array}{c} 0,\\ \left(\frac{EDWIN^{C}-EDWIN_{\min}^{c}}{EDWIN_{\max}^{C}-EDWIN_{\min}^{C}}\right)\\ 1, \end{array},\;\begin{array}{c} EDWIN^{C}\leq EDWIN_{\min}^{C}\\ EDWIN_{\min}^{C}\leq EDWIN^{C}\leq EDWIN_{\max}^{C}\\ EDWIN^{C}\geq EDWIN_{\max}^{C} \end{array}$$

(5) $$d_{2}=\{\begin{array}{c} 1,\\ \left(\frac{ST-ST_{\max}}{ST_{\min}-ST_{\max}}\right)\\ 0, \end{array},\;\begin{array}{c} ST_{1}\leq ST_{\min}\\ ST_{\min}\leq ST\leq ST_{\max}\\ ST\geq ST_{\max} \end{array}$$

For Equations (4) and (5), the bounds $ST_{\max}$ and $EDWIN_{\max}^{C}$ represented the upper specification limits; the bounds $ST_{\min}$ and $EDWIN_{\min}^{C}$ represented the lower specification limits. 

Table 1 lists several results for NN architecture options. To consider better performance with minimized training/testing RMSE (root of mean-square error), this study selected the structure 6-5-2 (input nodes–hidden nodes–output nodes). The structure 6-5-2 was well-trained by showing the function between factors levels and performances. This 6-5-2 was applied to forecast the two performances by inputting any combination of factor levels.

### 5.3. STEP 3. Use the GA to Obtain the Optimal Parameters Setting 

The GA, a solution method, was used here to derive the optimal parameters setting in the NN. The GA procedure is as follows:

The GA principle adopted the concept of “survival of the fittest,” which initially derived from “natural selection and genetics” developed by Darwin [55,56]. A GA is one of the soft computing techniques used as an optimization methodology to solve nonlinear programming problems. Unlike traditional selection techniques, setting the range of the feasible space and using a point-to-point search route to derive a solution, GA can be used to generate an optimal solution in the solution population through a series of iterative computations. Hence, GA can be used to deal with complicated problems in a pool of large search spaces to efficiently derive the optimal parameters solution. In GA, chromosomes are regarded as a set of alternative solutions to the problem, which consists of several genes, and are applied to derive an optimal solution; GA does not need to test all solutions. The main genetic operators, selection, crossover, and mutation, are used to converge the optimal solution by enhancing the fitness of a population of guesses. 

*TP* was set as $TP=\sqrt[{2}]{d_{1}*d_{2}}$ and as the total performance, which was also a GA fitness function. The control factor A was a continuous variable, and control factors B, C, and D were discrete variables. In addition, the operational conditions of the GA were set as follows: The number of generations was set to 1000. The population size was set to 80. The crossover rate was set to 0.5. The mutation rate was set to 0.08. The result showed that the optimal parameters setting for factors (A, B, C, D) was (Level 2.8, Level 1, Level 2, Level 2), and the total performance *TP* was 0.593.

### 5.4. STEP 4. Conduct Sensitivity Analysis for the EMS Adjustable Strategies

After the GA procedure, a sensitivity analysis for the parameters setting of the most robust levels, based on the results of the *TP*, was discussed. The sensitivity analysis was explored in the situation in which when one factors level changed, resulting in the change of other factor levels. The following results can be considered EMS adjustable strategies.

1. In Table 2, when factor A’s level changed from 2.8 to 1, the *TP* decreased from 0.529 to 0.593, and there was a 10.79% decrease in the adjusted *TP*%.
2. In Table 3, when factor B’s level was 2 or 3, the *TP* decreased to 0.558 or 0.516, and there was a 5.92% decrease or a 12.98% decrease in the adjusted *TP*%.
3. In Table 4, when factor C’s level was 1 or 3, the *TP* decreased to 0.559 or 0.562, and there was a 5.73% decrease or a 5.23% decrease in the adjusted *TP*%.
4. In Table 5, when factor D’s level was 1 or 3, the *TP* decreased to 0.576 or 0.590, and there was a 2.87% decrease or a 0.51% decrease in the adjusted *TP*%.

## 6. Conclusions

The development of GSCM in different industries has different motivations. For hospitals, the crucial motivation is to meet patients’ healthcare needs. Hence, the study used the concept of green innovation to design a better healthcare service flow to eliminate unnecessary healthcare waste and hence, increase healthcare quality. As Khan et al. [57] explained, an enterprise’s GSCM creates a positive impression for customers, leading to trust and satisfaction. 

This study used EDC as an example because EDC occurs when the demand for EMS is greater than the supply of EMS. The reasons behind this mismatch are complicated, including the general public’s incorrect view of emergency treatment, a shortage of manpower at EDs, limited space at EDs, a shortage of sickbeds, etc. These interlocking factors further increase the level of busyness at an ED and the workload of ED professionals, which affects patient wait time. Moreover, insufficient time or space to attend to patients leads to medical errors, worsening EDC.

The study used the dynamic Taguchi method to establish the EMS simulation system and to obtain the Taguchi’s parameters setting. The result of Taguchi parameters setting shows that based on $EDWIN^{C}$, the setting for the factors combination (A, B, C, D) was (Level 1, Level 1, Level 1, Level 1), and based on ST, the setting for the factors combination (A, B, C, D) was (Level 3, Level 3, Level 2, Level 2). Further, using the NN and GA to obtain the optimal parameters setting based on the *TP,* the setting for the factors combination (A, B, C, D) was (Level 2.8, Level 1, Level 2, Level 2). The sensitivity analysis shows the adjustable strategies. If factor A’s level moved from 2.8 to Level 1, Level 2, or Level 3, there was a 10.79%, 4.38%, or −0.33% decrease in the adjusted *TP*%. If factor B’s level moved from 1 to Level 2 or Level 3, there was a 5.92% or 12.98% decrease in the adjusted *TP*%. If factor C’s level moved from 2 to Level 1 or Level 3, there was a 5.73% or 5.23% decrease in the adjusted *TP*%. If factor D’s level moved from 2 to Level 1 or Level 3, there was a 2.87% or 0.51% decrease in the adjusted *TP*%.

The study makes two significant contributions. First, the study extended the significant influence of GSCM to hospitals to bring green innovation to hospitals. The core value of green innovation in the study aims to eliminate unnecessary healthcare waste to provide better quality healthcare. Eliminating waste improves the utilization of healthcare resources. Hospital administrators may hence design better GSCM activities to facilitate the healthcare process to provide better healthcare outcomes. 

The study’s second contribution was that it considered the adjustable number of physicians and EMS characteristics in the ED crowding problem. When the patients suddenly increase, the signal factor (the number of physicians) and the setting of control factors levels help stabilize the EMS treatment process. The dynamic Taguchi method can also be used in the GSCM to solve complex multi-performance problems. Nonetheless, most researchers have used the Taguchi method in other industries to improve products and processes through quality engineering. Future studies may use the Taguchi method combined with GA and NN to solve different departments’ multi-response optimization problems in the GSCM of healthcare systems.

GSCM emphasized the concept of reducing environmental impacts. The environmental impacts should include inter-organizational sharing responsibility [1,58]. Furthermore, GSCM is an interdisciplinary field which has a cross-function approach, involving internal activities and external activities [59]. Hence, GSCM should integrate internal activities and external activities to reduce environmental impacts. For most companies, to reduce environmental impacts is to reduce social loss (Taguchi loss function). Therefore, GSCM connected to the Taguchi loss function will be worth further discussion. As in this study, GSCM was applied in the EMS, which emphasizes the healthcare service quality for patients. Based on the Taguchi design, while selecting the service performances, the administrators should take the patients’ stay time in the systems and $EDWIN^{C}$ into consideration. For future study, GSCM connected to the Taguchi loss function (social loss) may be extended and applied to the service industries. 

As for the limitation of this study, the study used a medical center in central Taiwan as the object to set up the EMS simulation system to obtain a robust parameters setting. Hence, the results may not be extended to other hospitals with different sizes or different characteristics. To solve hospitals’ waste problems, those hospitals attempting to use the proposed mathematical model should select the parameters based on the characteristics of their hospitals to construct their own simulation scenario.

## Figures and Tables

**Table 1:** The neural network (NN) architecture options.

Structures (Input Nodes–Hidden Nodes–Output Nodes)	RMSE	
	Training	Testing
6-2-2	0.012169	0.019189
6-3-2	0.012117	0.018689
6-4-2	0.011963	0.018547
**6-5-2**	**0.011895**	**0.018524**
6-6-2	0.011970	0.018534
6-7-2	0.019823	0.019154
6-8-2	0.019983	0.019199

**Note**: The learning rate was set as auto-adjusting between 0.01 and 0.5; the momentum coefficient was 0.65; the number of iterations was 10,000; the bold numbers were the optimal structure.

**Table 2:** The values for the *TP* and the adjusted *TP*% resulting from a 0.2 level change in factor A.

Factor A	1	1.2	1.4	1.6	1.8	2
*TP*	0.529	0.536	0.546	0.550	0.557	0.567
Adjusted *TP*%	−10.79%	−9.61%	−7.92%	−7.25%	−6.07%	−4.38%
Factor A	2.2	2.4	2.6	2.8	3	
*TP*	0.573	0.581	0.589	**0.593**	0.591	
Adjusted *TP*%	−3.37%	−2.02%	−0.67%		−0.33%	

Adjusted *TP*% = $\frac{TP_{Level}-0.593}{0.593}$ where $TP_{Level}$ is the *TP* in factor A’s Level; the bold numbers were the optimal total performance.

**Table 3:** The values for the *TP* and the adjusted *TP*% resulting from a change in factor B.

Factor B	*1*	*2*	*3*
*TP*	**0.593**	0.558	0.516
Adjusted *TP*%		−5.92%	−12.98%

Adjusted *TP*% = $\frac{TP_{Level}-0.593}{0.593}$ where $TP_{Level}$ is the *TP* in factor B’s Level; the bold number was the optimal total performance.

**Table 4:** The values for the *TP* and the adjusted *TP*% resulting from a change in factor C.

Factor C	1	2	3
*TP*	0.559	**0.593**	0.562
Adjusted *TP*%	−5.73%		−5.23%

Adjusted *TP*% = $\frac{TP_{Level}-0.593}{0.593}$ where $TP_{Level}$ is the *TP* in factor C’s Level; the bold number was the optimal total performance.

**Table 5:** The values for the *TP* and the adjusted *TP*% resulting from a change in factor D.

Factor D	1	2	3
*TP*	0.576	**0.593**	0.590
Adjusted *TP*%	−2.87%		−0.51%

Adjusted *TP*% = $\frac{TP_{Level}-0.593}{0.593}$ where $TP_{Level}$ is the *TP* in factor D’s Level; the bold number was the optimal total performance.
